# Supplementary material for: A Bibenzyl from Dendrobium pachyglossum Exhibits Potent Anti-Cancer Activity Against Glioblastoma Multiforme
Source: Antioxidants (Basel). 2025 Oct 7;14(10):1212. doi: 10.3390/antiox14101212 (PMC12561214; doi:10.3390/antiox14101212)
Supplement: Supplementary file 1 [file antioxidants-14-01212-s001.zip › 250903 Supplementary Figures .pdf]

A Bibenzyl from *Dendrobium pachyglossum* Exhibits Potent Anti-Cancer Activity against Glioblastoma Multiforme

Hnin Mon Aung [1,2], Onsurang Wattanathamsan [2], Kittipong Sanookpan [2], Aphinan Hongprasit [2], Chawanphat Muangnoi [3], Rianthong Phumsuay [3], Thanawan Rojpitikul [1,4], Boonchoo Sritularak [4,5], Tankun Bunlue [6], Naphat Chantaravisoot [6,7,8], Claudia R. Oliva [9], Corinne E. Griguer [9], Visarut Buranasudja [1,2,5,*]

[1] Pharmaceutical Sciences and Technology Program, Faculty of Pharmaceutical Sciences, Chulalongkorn University, Bangkok, 10330, Thailand
[2] Department of Pharmacology and Physiology, Faculty of Pharmaceutical Sciences, Chulalongkorn University, Bangkok, 10330, Thailand
[3] Biological Science and Animal Model Unit, Institute of Nutrition, Mahidol University, Nakhon Pathom, 73170, Thailand
[4] Department of Pharmacognosy and Pharmaceutical Botany, Faculty of Pharmaceutical Sciences, Chulalongkorn University, Bangkok 10330, Thailand
[5] Center of Excellence in Natural Products for Ageing and Chronic Diseases, Faculty of Pharmaceutical Sciences, Chulalongkorn University, Bangkok, 10330, Thailand
[6] Department of Biochemistry, Faculty of Medicine, Chulalongkorn University, Bangkok, 10330, Thailand
[7] Center of Excellence in Systems Microbiology, Faculty of Medicine, Chulalongkorn University, Bangkok, 10330, Thailand
[8] Center of Excellence in Systems Biology, Faculty of Medicine, Chulalongkorn University, Bangkok, 10330, Thailand
[9] Free Radical & Radiation Biology Program, Department of Radiation Oncology, University of Iowa, IA, Iowa, 52242, USA

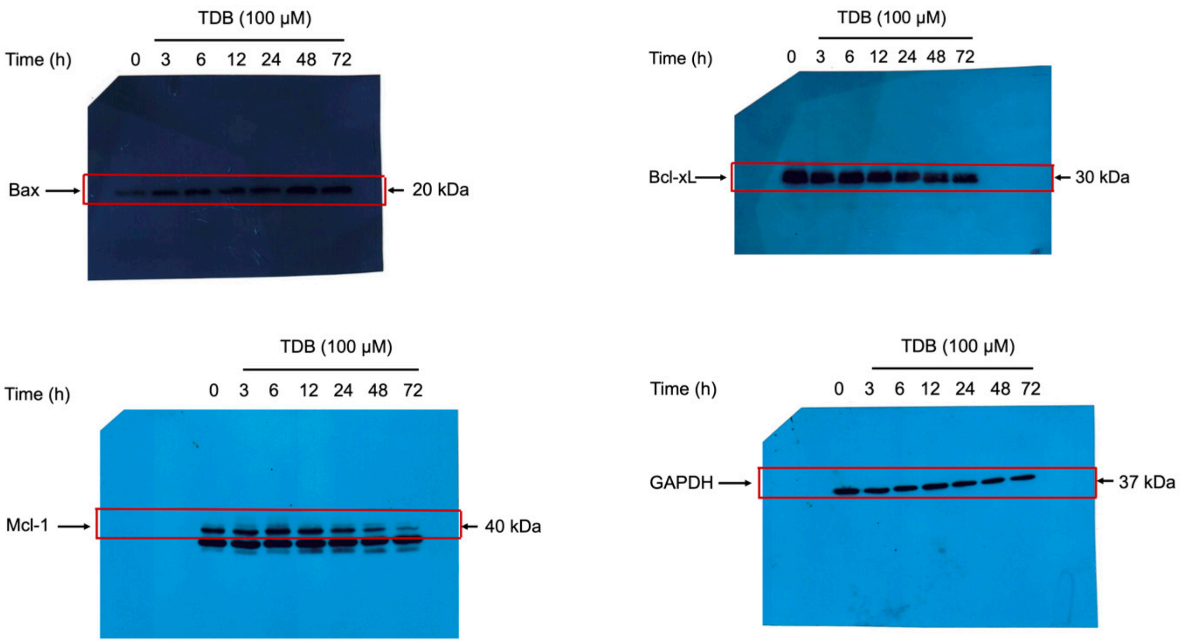

Supplementary Figure S1. Original unprocessed X-ray film of western blot corresponding to Figure 3C. The blot displays Bax, Bcl-xL, Mcl-1, and GAPDH bands, which were used for presentation in the main figure.

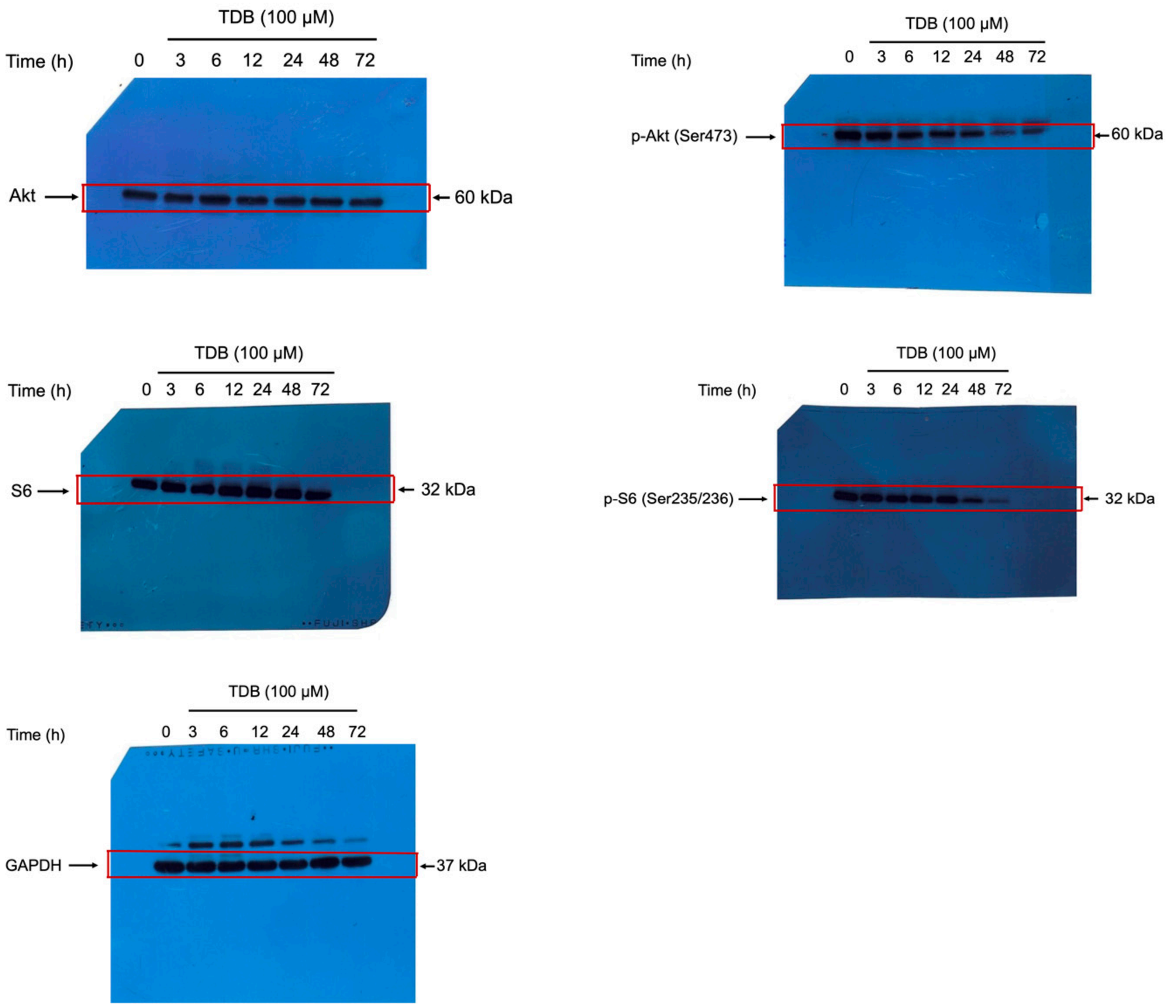

Supplementary Figure S2. Original unprocessed X-ray film of western blot corresponding to Figure 4A. The blot includes phosphorylated Akt (p-Akt), total Akt, phosphorylated S6 (p-S6), total S6, and GAPDH, which were displayed in the main figure.

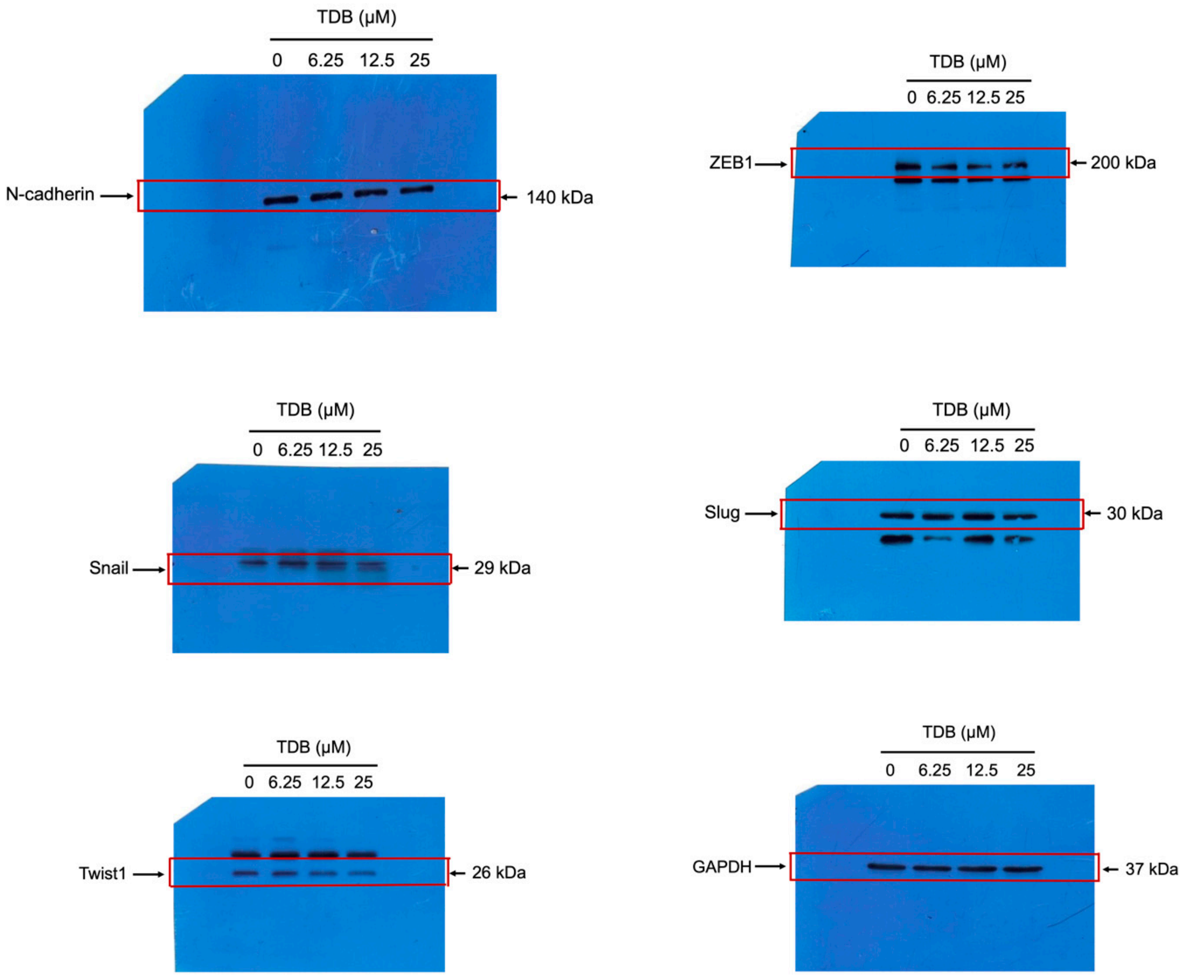

Supplementary Figure S3. Original X-ray film of western blot analysis corresponding to Figure 5C. The film displays EMT-related markers, and GAPDH displayed in main figure.

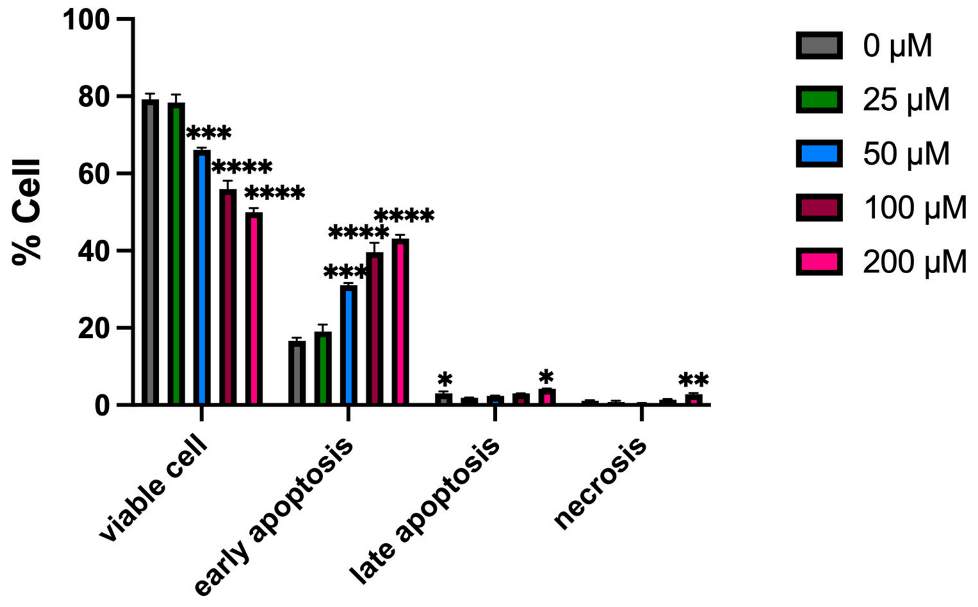

Supplementary Figure S4. Quantitative distribution of U87MG cell populations following TDB treatment. Cells were treated with various concentrations of TDB (25, 50, 100, and 200 μM) for 72 h, and apoptosis/necrosis was assessed by Annexin V-FITC and PI staining. Flow cytometric analysis shows the proportions of viable, early apoptotic, late apoptotic, and necrotic cells. Data represent the mean ± SEM from three independent biological replicates. * $p < 0.05$, ** $p < 0.01$, *** $p < 0.001$, and **** $p < 0.0001$ *v.s.* the untreated control.

4. Distribution

Property	Value	Decision	Comment
PPB	86.86	🟢	■ Plasma Protein Binding<br>Optimal: < 90%.<br>■ Drugs with high protein-bound may have a low therapeutic index.
VDss	-0.225	🔴	■ Volume Distribution<br>■ Optimal: 0.04-20L/kg
BBB	0.022	🟢	■ Blood-Brain Barrier Penetration<br>■ Category 1: BBB+; Category 0: BBB-;<br>■ The output value is the probability of being BBB+
Fu	14.491	🟢	■ The fraction unbound in plasms<br>■ Low: <5%; Middle: 5~20%; High: > 20%
OATP1B1 inhibitor	0.998	🔴	■ Category 0: Non-inhibitor; Category 1: inhibitor.<br>■ The output value is the probability of being inhibitor, within the range of 0 to 1.
OATP1B3 inhibitor	0.99	🔴	■ Category 0: Non-inhibitor; Category 1: inhibitor.<br>■ The output value is the probability of being inhibitor, within the range of 0 to 1.
BCRP inhibitor	0.688	🟠	■ Category 0: Non-inhibitor; Category 1: inhibitor.<br>■ The output value is the probability of being inhibitor, within the range of 0 to 1.
MRP1 inhibitor	0.71	🔴	■ Category 0: Non-inhibitor; Category 1: inhibitor.<br>■ The output value is the probability of being inhibitor, within the range of 0 to 1.

ADMETlab 3.0

Supplementary Figure S5. Distribution profile generated by the ADMETlab 3.0 platform predicting the blood–brain barrier (BBB) permeability of TDB. The output is presented within the distribution category, indicating its likelihood of crossing the BBB.

Result page displays query compound structures, predicted logBB values. ‘BBB Permeable’ or ‘BBB Non-Permeable’ is determined by the cutoff of logBB value. ‘BBB Permeable’ is LogBB >= -1.

SMILES	2D depiction	LogBB value	BBB Permeability
COc1cc(CCc2cc(O)c(O)c(OC)c2)ccc1O	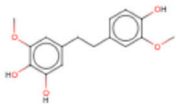	-0.42844	BBB Permeable

Supplementary Figure S6. Output generated by the LogBB_Pred model predicting the blood–brain barrier (BBB) permeability of TDB. The result page displays the SMILES representation, 2D chemical structure, predicted LogBB value, and the assigned BBB permeability classification.

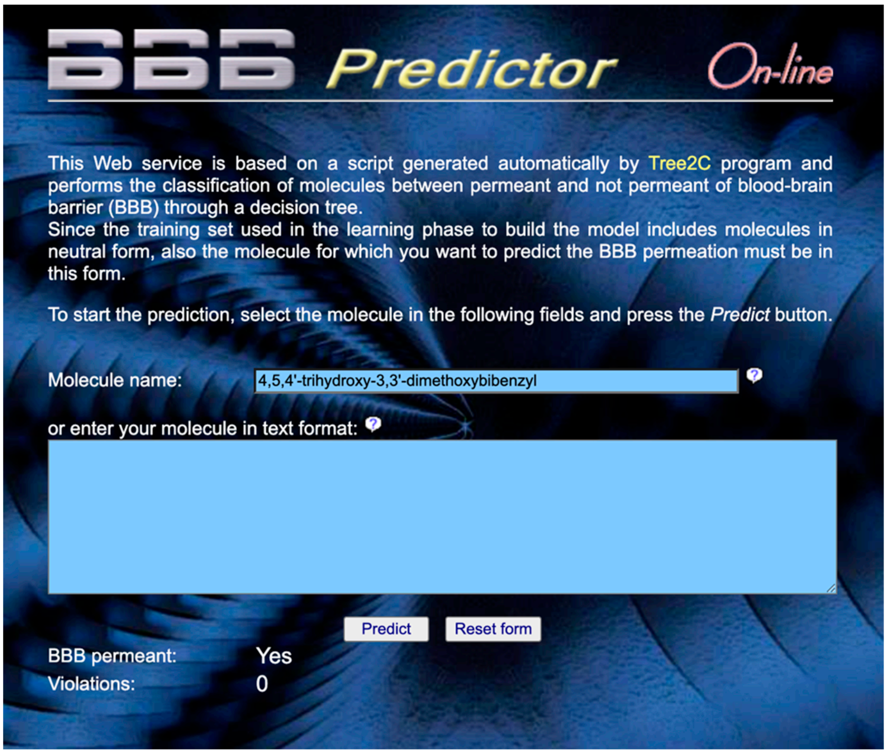

Supplementary Figure S7. Prediction output from the BBB Predictor (Tree2C) model for TDB. The interface displays the computational result indicating a positive classification for blood–brain barrier (BBB) permeability.

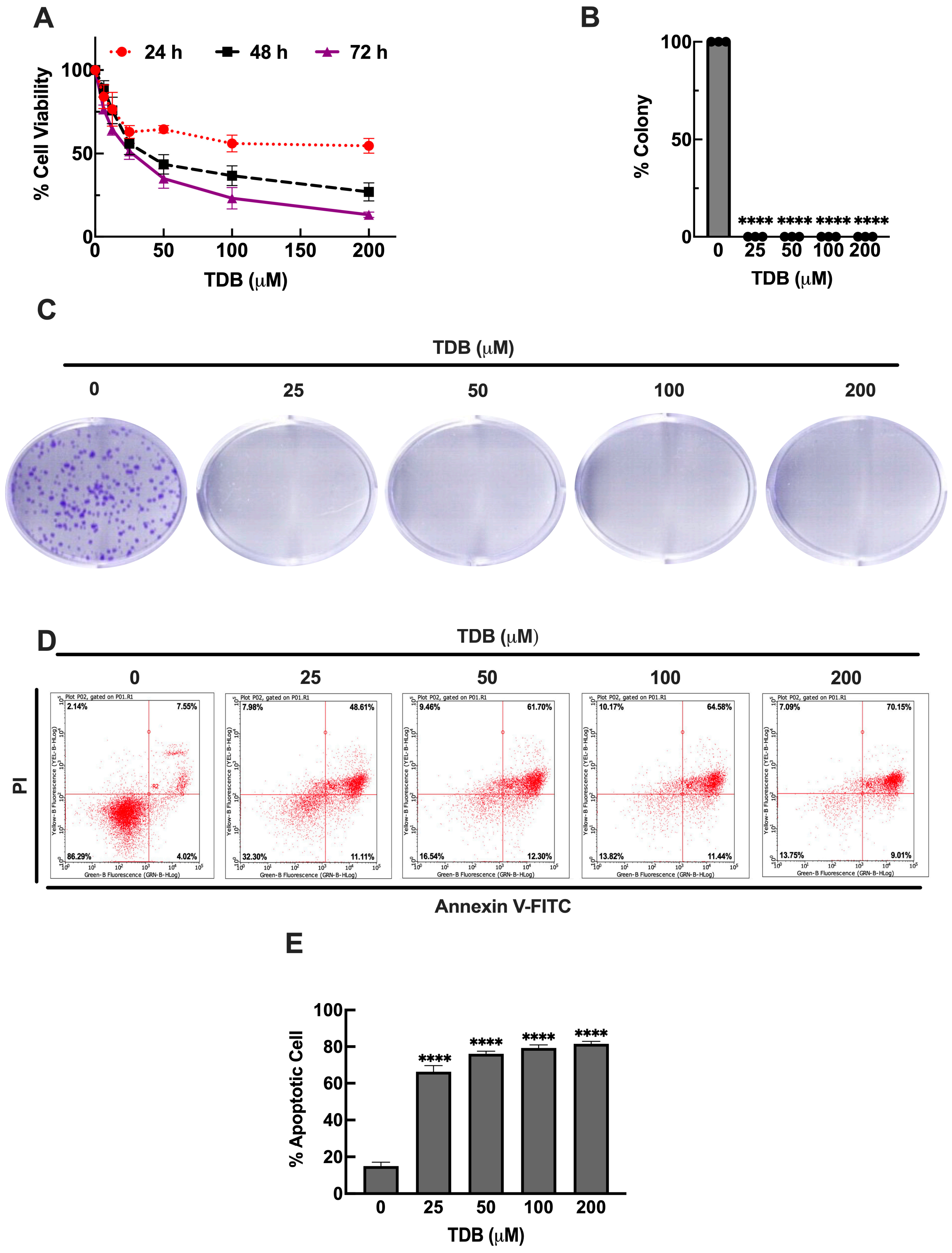

Supplementary Figure S8. Anticancer effects of TDB in low-grade glioma H4 cells.

(A) Cells were exposed to increasing concentrations of TDB (6.25 - 200 μM) for 24, 48, and 72 h, and cell viability was assessed using the MTT assay.

(B, C) Clonogenic survival was evaluated following 72 h of TDB treatment (25, 50, 100, and 200 μM), after which colonies were allowed to form and quantified.

(D, E) Apoptosis was analyzed after 72 h of TDB treatment (25, 50, 100, and 200 μM) using Annexin V-FITC/PI dual staining and flow cytometry. The apoptotic fraction includes both early and late apoptotic cells.

Data are presented as mean ± SEM from three independent experiments (*n* = 3). **** $p < 0.0001$ *v.s.* untreated control.

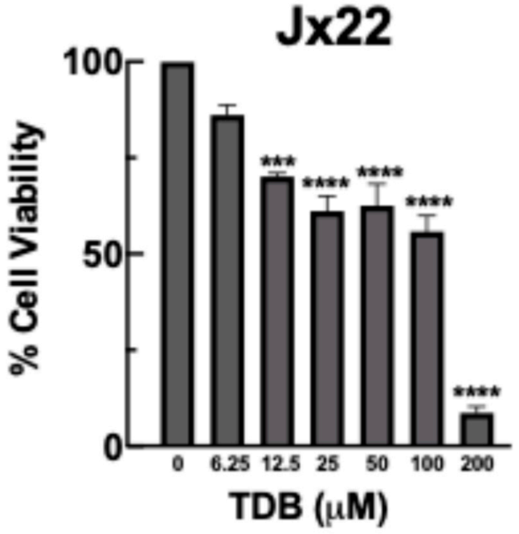

Supplementary Figure S9. Cytotoxic activity of TDB in patient-derived GBM Jx22 cells.

Cells were exposed to increasing concentrations of TDB (6.25–200 μM) for 72 h, and cell viability was assessed using the MTT assay. Data are presented as mean ± SEM from three independent experiments (n = 3). *** $p < 0.001$; **** $p < 0.0001$ *v.s.* untreated control.
