# Supplementary material for: A Bibenzyl from Dendrobium pachyglossum Exhibits Potent Anti-Cancer Activity Against Glioblastoma Multiforme
Source: Antioxidants (Basel). 2025 Oct 7;14(10):1212. doi: 10.3390/antiox14101212 (PMC12561214; doi:10.3390/antiox14101212)
Supplement: Supplementary file 1 [file antioxidants-14-01212-s001.zip › 250927 Supplementary Tables.pdf]

A Bibenzyl from *Dendrobium pachyglossum* Exhibits Potent Anti-Cancer Activity against Glioblastoma Multiforme

Hnin Mon Aung [1,2], Onsurang Wattanathamsan [2], Kittipong Sanookpan [2], Aphinan Hongprasit [2], Chawanphat Muangnoi [3], Rianthong Phumsuay [3], Thanawan Rojpitikul [1,4], Boonchoo Sritularak [4,5], Tankun Bunlue [6], Naphat Chantaravisoot [6,7,8], Claudia R. Oliva [9], Corinne E. Griguer [9], Visarut Buranasudja [1,2,5,*]

[1] Pharmaceutical Sciences and Technology Program, Faculty of Pharmaceutical Sciences, Chulalongkorn University, Bangkok, 10330, Thailand
[2] Department of Pharmacology and Physiology, Faculty of Pharmaceutical Sciences, Chulalongkorn University, Bangkok, 10330, Thailand
[3] Biological Science and Animal Model Unit, Institute of Nutrition, Mahidol University, Nakhon Pathom, 73170, Thailand
[4] Department of Pharmacognosy and Pharmaceutical Botany, Faculty of Pharmaceutical Sciences, Chulalongkorn University, Bangkok 10330, Thailand
[5] Center of Excellence in Natural Products for Ageing and Chronic Diseases, Faculty of Pharmaceutical Sciences, Chulalongkorn University, Bangkok, 10330, Thailand
[6] Department of Biochemistry, Faculty of Medicine, Chulalongkorn University, Bangkok, 10330, Thailand
[7] Center of Excellence in Systems Microbiology, Faculty of Medicine, Chulalongkorn University, Bangkok, 10330, Thailand
[8] Center of Excellence in Systems Biology, Faculty of Medicine, Chulalongkorn University, Bangkok, 10330, Thailand
[9] Free Radical & Radiation Biology Program, Department of Radiation Oncology, University of Iowa, IA, Iowa, 52242, USA

Supplementary Table S1. Detailed information on the chemicals and reagents used in this study.

Manufacturer	Chemicals	Catalogue number
Cell Signaling Technology (Danvers, MA, USA)	RIPA buffer (10X)	9806
	protease/phosphatase inhibitor cocktail (100X)	5872
Energenesis Biomedical (Taipei, Taiwan)	BlockPRO^{TM} 1 min blocking buffer	BM01-500
MilliporeSigma (Burlington, MA, USA)	bovine serum albumin	12659
	crystal violet	1159400100
	2,2-Diphenyl-1-picrylhydrazyl (DPPH)	D9132
	Ferric chloride hexahydrate	236489
	Immobilon western chemiluminescent HRP substrate	WBKLS0100
	2,4,6-Tris(2-pyridyl)-*s*-triazine (TPTZ)	T1253
Thermo Fisher Scientific (Waltham, MA, USA)	3-(4,5-Dimethyl-2-thiazolyl)-2,5-diphenyltetrazolium bromide (MTT)	M6494
	DMEM	12800-017
	DMEM/F12	12500-062
	Fetal bovine serum	A5256701
	Penicillin-streptomycin	15140-122
	Pierce BCA protein assay kit	23225
	Trypsin-EDTA	25200-072
Immuno Tools GmBH (Friesoythe, Germany)	Annexin V apoptosis detection kit FITC with PI	PI-3149001PI<br>Annexin V-31490013

Tokyo Chemical Industry (Tokyo, Japan)	temozolomide	T2744
HiMedia Laboratories (Maharashtra, India)	paraformaldehyde	GRM3660

Supplementary Table S2. List of antibodies used in western blot analysis. All antibodies were purchased from Cell Signaling Technology (Danvers, Massachusetts, USA).

Antibody	Source	Catalogue number	Concentration
Primary Antibody			
Akt	rabbit	9272	1:1000
p-Akt (Ser473)	rabbit	4060	1:2000
S6	rabbit	2217	1:1000
p-S6 (Ser235/236)	rabbit	4858	1:2000
Bax	rabbit	5023	1:1000
Bcl-xL	rabbit	2764	1:1000
Mcl-1	rabbit	94296	1:1000
N-cadherin	rabbit	13116	1:1000
ZEB1	rabbit	70512	1:1000
Snail	rabbit	3879	1:1000
Slug	rabbit	9585	1:1000
Twist1	rabbit	90445	1:1000
GAPDH	rabbit	5174	1:1000
Secondary Antibody			
Anti-rabbit IgG, HRP-linked antibody	goat	7074	1:2000

Supplementary Table S3. The *p*-values corresponding to each graph presented in the main manuscript. Unless otherwise specified, *p*-values represent comparisons versus the untreated control group.

**Figure**	**Experimental Condition**	***p value***
2B	TDB 25 μM	0.0032
	TDB 50 μM	0.0001
	TDB 100 μM	<0.0001
	TDB 200 μM	<0.0001
3B	TDB 25 μM	0.9569
	TDB 50 μM	0.0004
	TDB 100 μM	<0.0001
	TDB 200 μM	<0.0001
3D (Bax)	3 h	0.2556
	6 h	0.0700
	12 h	0.0736
	24 h	0.0806
	48 h	0.0207
	72 h	0.0301
3D (Bcl-xL)	3 h	0.0803
	6 h	0.1295
	12 h	0.3227
	24 h	0.0578
	48 h	0.0041
	72 h	<0.0001
3D (Mcl-1)	3 h	0.8370
	6 h	0.0633
	12 h	0.4412
	24 h	0.3924
	48 h	0.0308
	72 h	0.0006

Supplementary Table S3. (Continued)

Figure	Experimental Condition	*p value*
4B (Akt)	3 h	0.3106
	6 h	0.2645
	12 h	0.8579
	24 h	0.5484
	48 h	0.6604
	72 h	0.0950
4B (p-Akt)	3 h	0.0358
	6 h	0.0115
	12 h	0.0131
	24 h	0.0019
	48 h	0.0139
	72 h	0.0006
4B (S6)	3 h	0.5239
	6 h	0.4896
	12 h	0.5817
	24 h	0.4524
	48 h	0.9491
	72 h	0.3166
4B (p-S6)	3 h	0.2938
	6 h	0.8016
	12 h	0.8330
	24 h	0.2465
	48 h	0.0075
	72 h	0.0002
5D (N-Cadherin)	TDB 6.25 μM	0.0032
	TDB 12.5 μM	0.0011
	TDB 25 μM	0.0089
5D (ZEB1)	TDB 6.25 μM	0.2249
	TDB 12.5 μM	0.3912
	TDB 25 μM	0.0070
5D (Snail)	TDB 6.25 μM	0.9739
	TDB 12.5 μM	0.0826
	TDB 25 μM	0.0492

Supplementary Table S3. (Continued)

**Figure**	**Experimental Condition**	***p value***
5D<br>(Slug)	TDB 6.25 µM<br>TDB 12.5 µM<br>TDB 25 µM	0.3133<br>0.4808<br>0.0016
5D<br>(Twist1)	TDB 6.25 µM<br>TDB 12.5 µM<br>TDB 25 µM	0.3041<br>0.0051<br>0.0009
6A	TMZ 2.5 µM<br>TMZ 5 µM<br>TMZ 10 µM<br>TMZ 25 µM<br>TMZ 50 µM	0.0224<br>0.0048<br>0.0026<br><0.0001<br><0.0001
6C<br>U87MG	TMZ<br>TMZ + TDB 25 µM<br>TMZ + TDB 50 µM	0.0004<br><0.0001<br><0.0001
6C*<br>U87MG<br>(*v.s.* TMZ)	TMZ + TDB 25 µM<br>TMZ + TDB 50 µM	0.0155<br>0.0073
6D<br>Jx22	TMZ<br>TMZ + TDB 25 µM<br>TMZ + TDB 50 µM	0.0005<br><0.0001<br><0.0001
6D*<br>Jx22<br>(*v.s.* TMZ)	TMZ + TDB 25 µM<br>TMZ + TDB 50 µM	0.0003<br>0.0002
